# Telehealth and Palliative Care for Patients With Cancer: Implications of the COVID-19 Pandemic

**DOI:** 10.2196/20288

**Published:** 2020-11-24

**Authors:** Udhayvir Singh Grewal, Stephanie Terauchi, Muhammad Shaalan Beg

**Affiliations:** 1 Department of Internal Medicine Louisiana State University Health Sciences Center Shreveport, LA United States; 2 Department of Internal Medicine University of Texas Southwestern Medical Center Dallas, TX United States; 3 Simmons Comprehensive Cancer Center University of Texas Southwestern Medical Center Dallas, TX United States

**Keywords:** COVID-19, telehealth, palliative care, telepalliative care, patients with cancer, telemedicine

## Abstract

It has been reported that the incidence of SARS-CoV-2 infection is higher in patients with cancer than in the general population and that patients with cancer are at an increased risk of developing severe life-threatening complications from COVID-19. Increased transmission and poor outcomes noted in emerging data on patients with cancer and COVID-19 call for aggressive isolation and minimization of nosocomial exposure. Palliative care and oncology providers are posed with unique challenges due to the ongoing COVID-19 pandemic. Telepalliative care is the use of telehealth services for remotely delivering palliative care to patients through videoconferencing, telephonic communication, or remote symptom monitoring. It offers great promise in addressing the palliative and supportive care needs of patients with advanced cancer during the ongoing pandemic. We discuss the case of a 75-year-old woman who was initiated on second-line chemotherapy, to highlight how innovations in technology and telehealth-based interventions can be used to address patients’ palliative and supportive care needs in the ongoing epidemic.

## Cancer and COVID-19

A nationwide analysis from China has indicated that the incidence of SARS-CoV-2 infection is higher in patients with cancer than in the general population and that patients with cancer are at an increased risk of developing severe life-threatening complications from COVID-19. Compared to patients without cancer, patients with cancer are about 3.5 times more likely to be admitted to the intensive care unit or die due to complications of COVID-19 [1]. The increased risk of infection and complications in these patients could be due to immunosuppression caused by the cancer itself or myelosuppression secondary to treatment, such as chemotherapy [2]. Due to the increased risk of complications in patients with cancer, there is growing concern about these patients experiencing delays in the delivery of necessary care and medical services, including palliative and supportive care [3]. Palliative medicine is a vital component of cancer care, and it can be provided in a variety of clinical settings, including outpatient clinics, inpatient consultations, dedicated palliative medicine units, long-term care facilities, and home-based care. Although inpatient palliative medicine is usually more common in practice, the delivery of outpatient palliative care has been growing, and it has been shown to improve the quality of life and overall well-being of patients with cancer [4].

Palliative care and oncology providers are posed with unique challenges due to the ongoing COVID-19 pandemic. Increased transmission and poor outcomes noted in emerging data on patients with cancer and COVID-19 call for aggressive isolation and minimization of nosocomial exposure [1]. Health care providers are challenged to innovate and develop care delivery systems that can balance the benefits of the care delivered with the risk and burden posed to patients by exposure to health care personnel. The decision to treat patients and risk exposure and infection-related complications needs to be weighed against the risk that is posed by the delay in treatments. Providing palliative care to cancer survivors during the ongoing pandemic may be as daunting a challenge as those presented by therapeutic dilemmas.

## Telehealth and Telepalliative Care

Telehealth has been defined by the US Health Resources and Service Administration as “the use of electronic information and telecommunications technologies to support long-distance clinical health care, patient and professional health-related education, public health and health administration” [5]. Several studies have suggested that telehealth is not only cost-effective, but also associated with encouragingly high levels of patient satisfaction [6-8]. Telehealth services have helped bridge travel-related barriers in a cost-effective manner. The implementation of telehealth-based interventions has also been associated with improved overall outcomes, such as improved medication compliance rates and shorter hospital stays [9]. As the number of COVID-19 cases continues to rise across the world, health care systems have been adopting virtual treatment options to minimize the need for physical meetings between patients and health care providers. This has been considered the new normal for both physicians and patients. Virtual care has shown promise in terms of reducing the number of emergency room visits, conserving health care resources, and minimizing the spread of COVID-19 [10].

Telepalliative care is the use of telehealth services for remotely delivering palliative care to patients through videoconferencing, telephonic communication, or remote symptom monitoring. Although careful planning is needed to set up and implement a robust system for its delivery, telepalliative care has been widely accepted by patients, and it can be used for various patient populations, including patients who are very susceptible to infection [11].

Let us consider the case of a patient in need of palliative care in the era of COVID-19.

Ms Smith is a 75-year-old woman with metastatic pancreatic cancer and worsening back pain. She has considered starting second-line chemotherapy, and has been referred to a palliative care program. Ms Smith lives alone, and a neighbor drives her to and from the clinic on chemotherapy days. Due to the COVID-19–related reduction in clinical staff, palliative care clinical services have been reduced. The next available clinic encounter is in 14 days.

## How Can Technology and Telehealth-Based Services be Used to Meet Ms Smith’s Palliative Care Needs in Light of the Ongoing Pandemic?

Studies have shown that virtual visits do not compromise the quality of care and are as effective as in-person visits for delivering palliative care [12]. Videoconferencing can be a valid tool for Ms Smith’s initial assessment. In our experience, apps embedded in electronic health records (eg, Epic, MyChart, and BlueJeans) and free-standing apps (eg, Doximity Dialer) can be effective modes of virtual communication between patients and clinicians. Audio-visual platforms for videoconferencing provide an opportunity for palliative care physicians to interact with patients, obtain a medical history, and assess current symptom burden. Physicians can use a virtual physical exam, supplemented by patient self-examination, to augment their clinical assessment. These measures can be used to estimate the current functional status of the patient and evaluate certain physical characteristics, such as vital signs, general physical appearance, cardiorespiratory status, changes in skin and extremities, and changes in performance status [13].

During an initial virtual visit, the palliative care provider can address the patient’s pain, which is a common symptom experienced by patients with cancer. Managing pain for a patient with cancer is a significant challenge, and it has a significant impact on patients’ overall outcomes. Uncontrolled pain can lead to hospitalization, which can increase the chance of COVID-19 exposure [14]. Effective communication between patients and their palliative care team can improve pain management and patient satisfaction [15]. Furthermore, the efficacy of patient-physician interactions via videoconferencing is comparable to that of in-person evaluation and care [16]. Patient history and virtual physical exam data augmented with data from patient self-examination can help physicians make an accurate assessment of the patient’s pain [17]. Knowing the location and nature of the pain, aggravating and relieving factors, and relationship between pain and posture can help physicians determine the etiology of pain and inform the subsequent management.

Although telehealth can be an effective way to manage pain and other aspects of patient care, prior legislation prohibited health care providers from prescribing opioids solely through telehealth services. However, because the COVID-19 pandemic has been declared a national emergency, the United States Drug Enforcement Administration, under the conditions outlined in the Ryan Haight Act (Title 21, United States Code, Section 802[54][D]), has allowed physicians to prescribe controlled substances (schedule II-IV) through telehealth services, even for patients that physicians have not evaluated in person [18]. This change in legislation has removed pre-existing barriers and has allowed physicians to continue to provide pain and symptom management to those who need it most.

Depression, anxiety, and psychological distress are important issues that affect a significant proportion of patients with cancer, especially those with metastatic disease. Virtual visits may also be used as an opportunity to identify psychological distress and emotional stressors. Telepsychiatry-based interventions have been increasingly incorporated into mainstream practice and have shown accurate results and overall outcomes comparable to those of in-person interventions [19].

Virtual visits can also be used as an opportunity for advance care planning with the patient. Per the White House Coronavirus Task Force, epidemiological models have predicted about 100,000 deaths associated with COVID-19 in the United States alone [20]. These estimates call for timely advance care planning with patients at a higher risk of mortality due to COVID-19, such as patients with cancer. Therefore, regular telehealth visits with patients with cancer should also focus on advance care planning, specifically in reference to the ongoing pandemic. Proactive discussions with patients about their health care expectations and goals would facilitate the appropriate delivery of care to these patients, should they contract COVID-19 and develop serious complications [21]. These conversations are intimate and potentially emotional for patients with cancer. Telehealth services, such as videoconferencing, help maintain a personal connection by allowing physicians to engage with and be responsive to their patients’ cues. The clinician should maintain the best possible environment for consultations and ask for the patient’s permission before beginning a consultation. Acknowledging the patient’s emotions and providing defined pauses to allow patients to reflect on, summarize, and repeat information are important when providing a virtual consultation.

For subsequent patient monitoring and ongoing palliative care delivery, telemonitoring or home-based telehealth services can be employed. These also allow for the remote monitoring of symptoms after the patient starts systemic chemotherapy. Electronic telehealth-based tools can be used for the remote symptom management of the patient. A questionnaire based on the different symptoms of chemotherapy-associated toxicity can be self-administered by the patient and recorded via a remote mobile phone. The results of the questionnaire can be used to automatically generate advice for managing the patient’s symptoms. This advice is then sent to the patient’s remote device. If the symptoms are significantly concerning, then a notification can be sent to the physician’s handset, prompting an appropriate response [22]. Therefore, continuous telemonitoring allows for regular checks on the functional status and general well-being of the patient. It can also alert providers to significant events, such as the development of serious adverse effects associated with chemotherapy.

Alerting clinicians to major changes in patient-reported outcomes can allow health care providers to intervene early by managing treatment-related side effects before they cause complications. This provides an opportunity to use outpatient services or arrange direct admissions to the hospital for fluid resuscitation or pain control, thereby preventing the need for emergency department visits. Patients who do not require immediate hospitalization or dedicated medical care after discharge can be considered for home-based health care, along with telepalliative care for symptom management.

**Figure 1:**
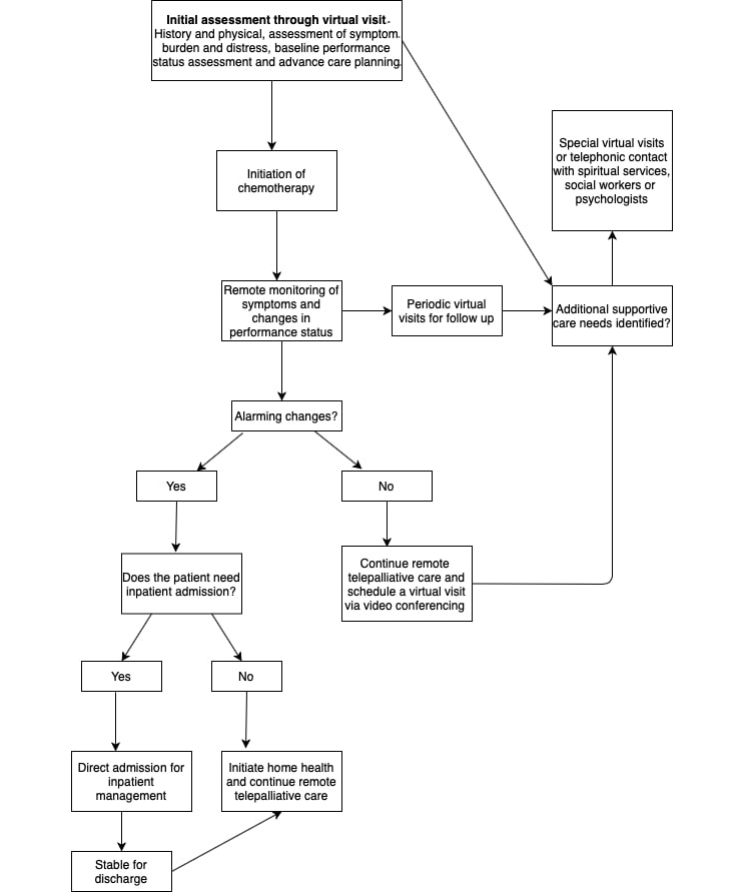
Flowchart demonstrating a telepalliative plan of care for patients with metastatic cancer who are initiated on second-line therapy.

Follow-up virtual visits can be conducted via videoconferencing. During these visits, the need for special medical or additional supportive care can be addressed. Telepalliative care allows for continued multidisciplinary management and addresses a patient’s well-being. In a time when the feeling of uncertainty is high, access to a multidisciplinary supportive care team can help with the emotional well-being of patients and their family. Figure 1 illustrates a telepalliative care-based plan for patients. Visits with spiritual care providers, social workers, and psychologists can be conducted through telehealth services.

## Conclusion

Telepalliative care offers great promise in addressing the palliative and supportive care needs of patients with advanced cancer during the ongoing pandemic. Continuous telemonitoring can be used to remotely monitor crucial patient-reported outcomes, such as pain and respiratory distress. Periodic virtual visits can provide oncology and palliative care providers the opportunity to address additional care needs and assess alarming changes that warrant hospitalization. However, the implementation of telepalliative care is limited by several barriers, such as limited remuneration by insurance agencies and poor access for communities with limited internet access. Various state-specific regulations and strict requirements for medical licensure and credentialing would also geographically limit the delivery of telepalliative care [23]. The ripple effect of COVID-19 will outlast the pandemic itself, and the impacts of this ripple effect on the health care delivery system and health care for patients with cancer will last longer. It is important to devise strategies for delivering effective palliative care to patients with advanced cancer. Telehealth-based interventions offer promise for the remote delivery of palliative care and effective symptom management. Telehealth and technology services should be implemented in clinical practice in a sustainable and patient-centric manner.
